# Conditional *Oprk1*-dependent *Kiss1* deletion in kisspeptin neurons caused estrogen-dependent LH pulse disruption and LH surge attenuation in female rats

**DOI:** 10.1038/s41598-023-47222-5

**Published:** 2023-11-22

**Authors:** Mayuko Nagae, Koki Yamada, Yuki Enomoto, Mari Kometani, Hitomi Tsuchida, Arvinda Panthee, Miku Nonogaki, Nao Matsunaga, Marina Takizawa, Sena Matsuzaki, Masumi Hirabayashi, Naoko Inoue, Hiroko Tsukamura, Yoshihisa Uenoyama

**Affiliations:** 1 Laboratory of Animal Reproduction, Graduate School of Bioagricultural Sciences, Nagoya University, Nagoya, Aichi 464-8601 Japan; 2 Section of Mammalian Transgenesis, Center for Genetic Analysis of Behavior, National Institute for Physiological Sciences, Okazaki, Aichi 444-8787 Japan

**Keywords:** Neuroscience, Reproductive biology

## Abstract

The gonadotropin-releasing hormone (GnRH) pulse and surge are considered to be generated by arcuate kisspeptin/neurokinin B/dynorphin A (KNDy) neurons and anteroventral periventricular nucleus (AVPV) kisspeptin neurons, respectively, in female rodents. The majority of KNDy and AVPV kisspeptin neurons express κ-opioid receptors (KORs, encoded by *Oprk1*) in female rodents. Thus, this study aimed to investigate the effect of a conditional *Oprk1*-dependent *Kiss1* deletion in kisspeptin neurons on the luteinizing hormone (LH) pulse/surge and fertility using *Kiss1*-floxed/*Oprk1-Cre* rats, in which *Kiss1* was deleted in cells expressing or once expressed the *Oprk1/Cre*. The *Kiss1*-floxed/*Oprk1-Cre* female rats, with *Kiss1* deleted in a majority of KNDy neurons, showed normal puberty while having a one-day longer estrous cycle and fewer pups than *Kiss1*-floxed controls. Notably, ovariectomized (OVX) *Kiss1*-floxed/*Oprk1-Cre* rats showed profound disruption of LH pulses in the presence of a diestrous level of estrogen but showed apparent LH pulses without estrogen treatment. Furthermore, *Kiss1*-floxed/*Oprk1-Cre* rats, with *Kiss1* deleted in approximately half of AVPV kisspeptin neurons, showed a lower peak of the estrogen-induced LH surge than controls. These results suggest that arcuate and AVPV kisspeptin neurons expressing or having expressed *Oprk1* have a role in maintaining normal GnRH pulse and surge generation, the normal length of the estrous cycle, and the normal offspring number in female rats.

## Introduction

Reproductive function is precisely orchestrated by hypothalamic neuropeptides, namely kisspeptin (encoded by the *Kiss1* gene) and gonadotropin-releasing hormone (GnRH), pituitary gonadotropins, and gonadal steroids in mammals^1–4^. There are tonic (pulsatile) or cyclic (surge) modes of GnRH release, which are responsible for folliculogenesis/steroidogenesis or ovulation, respectively, in female mammals^1–4^. The GnRH pulse is fundamentally important for tonic gonadotropin release because pulsatile GnRH treatment at the physiological frequency restores gonadotropin release in female rhesus monkeys with hypothalamic lesions, whereas continuous administration of GnRH paradoxically suppresses gonadotropin release^5^. Recently, we have provided direct evidence that KNDy (an acronym for kisspeptin/neurokinin B [NKB]/dynorphin A [Dyn]) neurons in the arcuate nucleus (ARC) serve as the GnRH pulse generator by showing that the rescue of KNDy neurons by transfecting the *Kiss1* gene into ARC NKB neurons recovered luteinizing hormone (LH) pulses and folliculogenesis in infertile global *Kiss1* knockout (KO) female rats^6^. Recent in vivo Ca^2+^ imaging also showed synchronized rhythmic increases in intracellular Ca^2+^ levels in clusters of KNDy neurons, and rhythmic increases in intracellular Ca^2+^ levels in KNDy neurons were accompanied by LH pulses in male and female mice^7–9^. On the other hand, the GnRH surge, which is evoked by the positive feedback action of estrogen derived from mature follicles, is crucially important for the LH surge and ovulation in mammals^1^. Kisspeptin neurons located in the anteroventral periventricular nucleus (AVPV) serve as a target of estrogen positive feedback action on GnRH/LH surge generation in rodents because estrogen increases AVPV *Kiss1* expression or c-Fos (a marker of neuronal activation) in AVPV kisspeptin neurons in female rats^10–12^ and mice^13–15^. The notion that AVPV kisspeptin neurons serve as the GnRH surge generator has also been verified by sex differences in the numbers of AVPV kisspeptin neurons (males < females) and the absence of an estrogen-induced LH surge in male rodent models^4, 11, 16–19^. Importantly, Wiegand et al.^20^ demonstrated that AVPV lesion disrupted estrogen + progesterone-induced LH surge in female rats. Furthermore, our and other previous studies showed that kisspeptin antagonism by chronic intracerebroventricular infusion of a GPR54 antagonist or by chronic infusion of an anti-kisspeptin antibody into the preoptic area blocked spontaneous or estrogen-induced LH surge in female rats^10, 21, 22^.

It has been suggested that KNDy neurons synchronize with each other in a paracrine and autocrine manner through stimulatory NKB-neurokinin 3 receptor (NK3R, the primary receptor for NKB) signaling and inhibitory Dyn-κ-opioid receptor (KOR, the primary receptor for Dyn) signaling to form GnRH/gonadotropin pulses because the majority of KNDy neurons express NK3Rs and KORs in rats and sheep^23–25^. Indeed, central administration of NKB or a KOR antagonist increased the frequency of multiple unit activity (MUA) volleys accompanied by LH pulses, and central Dyn administration decreased the frequency of MUA volleys in female goats^26^, in which MUA volleys were taken from the cluster of KNDy neurons. In addition, Ruka et al.^27^ and de Croft et al.^28^ showed that an NK3R agonist increased and Dyn or a KOR agonist decreased the firing frequency of GFP-labeled KNDy neurons in male mice. Moreover, the LH pulse frequency was increased by peripheral administration of the KOR antagonist and was suppressed by peripheral administration of the NK3R antagonist in female goats^29^ and ewes^30^. Importantly, KOR expression was found in some KNDy neurons in rats (62%)^25^, sheep (98%)^24^, and mice (6–65%)^27, 31–33^, but it has not been clarified whether KOR-expressing KNDy neurons play an indispensable role in fertility and GnRH pulse generation. Therefore, it is worth clarifying the effects of a conditional *Oprk1*-dependent *Kiss1* deletion in KNDy cells on fertility and LH pulses in newly generated gene-modified female rats.

The sex steroidal milieu should be taken into account when studying the role of Dyn-KOR signaling in GnRH pulse generation by KNDy neurons because Dyn-KOR signaling mediates the negative feedback action of sex steroids and stress-induced suppression of GnRH/gonadotropin pulses in several mammals, including rodents, ruminants, primates, and humans^34^. Indeed, Han et al.^35^ showed that central KOR antagonism promoted synchronization between KNDy neurons in testis-intact *Kiss1-Cre* male mice, but not in castrated males. Furthermore, our recent study showed that central administration of a KOR antagonist restored glucoprivic suppression of LH pulses in ovariectomized rats treated with a diestrous level of estradiol-17β (OVX + low E2 rats)^25^. Interestingly, low E2 treatment significantly increased the number of *Pdyn* (encoding Dyn)-expressing cells in the paraventricular nucleus (PVN) but not in the ARC of OVX rats^25^, suggesting that PVN Dyn neurons mainly mediate the inhibitory effect of sex steroids and/or malnutrition on KNDy neural activity and GnRH/LH pulses. Therefore, the contribution of Dyn-KOR signaling in controlling pulsatile GnRH/LH release could be changed depending on the steroidal milieu.

It is also known that KORs are expressed in some (50%) AVPV kisspeptin neurons in mice^32^, while the role of Dyn-KOR signaling in AVPV kisspeptin neurons has not yet been clarified. If KOR-expressing AVPV kisspeptin neurons are needed for GnRH/LH surge generation, a conditional *Oprk1*-dependent *Kiss1* deletion in AVPV kisspeptin cells may affect the LH surge and ovulation.

**Figure 1:**
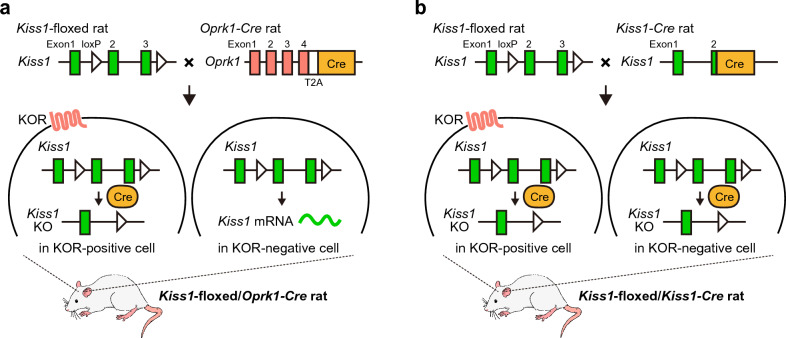
Schematic illustration of conditional *Kiss1* knockout (KO) in κ-opioid receptor (KOR)-positive or all kisspeptin neurons in female rats. (**a**) Generation of rats with conditionally deleted *Kiss1* in KOR-positive anteroventral periventricular nucleus (AVPV) and arcuate nucleus (ARC) kisspeptin neurons (*Kiss1*-floxed/*Oprk1-Cre* rats) by crossing *Oprk1-Cre* rats with *Kiss1*-floxed rats. (**b**) Generation of rats with conditionally deleted *Kiss1* in all kisspeptin neurons (*Kiss1*-floxed*/Kiss1-Cre* rats) by crossing *Kiss1-Cre* rats with *Kiss1*-floxed rats.

Thus, the present study aimed to investigate the effect of a conditional *Oprk1*-dependent *Kiss1* deletion in kisspeptin cells on LH pulses, the LH surge, and fertility using newly generated *Kiss1*-floxed/*Oprk1-Cre* rats (Fig. 1a) to clarify whether KOR-negative kisspeptin neurons could maintain fertility and whether KOR-positive kisspeptin neurons are a prerequisite for the pulsatile and surge modes of GnRH/LH release. Puberty onset, ovarian weight, vaginal cyclicity, and fertility were examined in ovary-intact *Kiss1*-floxed/*Oprk1-Cre* rats and *Kiss1*-floxed control rats. Furthermore, ARC *Kiss1* mRNA expression; pulsatile LH release; and pituitary *Gnrhr*, *Lhb*, and *Fshb* mRNA (encoding GnRH receptor, and LH and FSH β-subunit, respectively) expression were examined in *Kiss1*-floxed/*Oprk1-Cre* rats and *Kiss1*-floxed control rats under different steroidal milieus (OVX vs. physiological estrogenic condition, i.e., OVX + low E2 condition). In addition, we examined effects of the conditional *Kiss1* deletion on the number of AVPV *Kiss1*-expressing cells and on the LH surge induced by a proestrous level of E2 (high E2) by comparing *Kiss1*-floxed/*Oprk1-Cre* rats and *Kiss1*-floxed control rats. Moreover, for comparison with *Kiss1*-floxed/*Oprk1-Cre* rats, we generated rats with conditionally deleted *Kiss1* in all kisspeptin neurons (*Kiss1*-floxed/*Kiss1-Cre* rats) by crossing *Kiss1*-*Cre* rats^36^ with *Kiss1*-floxed rats (Fig. 1b), and reproductive indicators, such as puberty onset, ovarian weight, ARC *Kiss1* mRNA expression, pulsatile LH release, and pituitary *Gnrhr*, *Lhb*, and *Fshb* mRNA expression, were also examined in the *Kiss1*-floxed/*Kiss1-Cre* rats.

## Results

### Colocalization of *Oprk1* and *Oprk1-Cre*-activated *tdTomato* mRNA expression

We first generated *Oprk1-Cre* rats (see Supplementary Fig. S1 online) and confirmed the specificity of *Cre* expression in *Oprk1*-expressing cells by crossing *Oprk1-Cre* rats and Cre-dependent tdTomato reporter rats^37^ (see Supplementary Fig. S2a online). Double in situ hybridization (ISH) revealed the presence of a number of *Oprk1*-expressing cells and *tdTomato*-expressing cells in the hypothalamus, including the ARC (Fig. S2b), PVN (Fig. S2c), and supraoptic nucleus (SON, Fig. S2d), in OVX + low E2 *Oprk1-Cre*/tdTomato reporter female rats in adulthood. Quantitative analysis revealed that the majority of *Oprk1*-expressing cells coexpressed *tdTomato* in the ARC (92.96 ± 1.35%), PVN (98.06 ± 0.35%), and SON (97.95 ± 0.40%) in *Oprk1-Cre*/tdTomato reporter rats, indicating that most *Oprk1*-expressing cells in these regions were labeled with the *tdTomato* reporter (Fig. S2b-d). On the other hand, many *tdTomato*-expressing cells without *Oprk1* expression were observed in these regions because theoretically cells that have expressed *Oprk1*/*Cre* at least once should exhibit persistent *tdTomato* expression after *Oprk1*/*Cre* expression in *Oprk1-Cre*/tdTomato reporter rats. Quantitative analysis revealed that 31.16 ± 3.75% (ARC), 35.63 ± 5.72% (PVN), and 46.07 ± 8,68% (SON) of *tdTomato*-expressing cells coexpressed *Oprk1*.

### *Oprk1*-driven Cre-activated *tdTomato* expression in KNDy neurons in female rats at prepubertal and adult stages

Double ISH for *tdTomato* and *Tac3* (encoding NKB, a marker of KNDy neurons) revealed *Oprk1-Cre*-activated *tdTomato* expression in a small population of ARC *Tac3*-expressing cells (19.48 ± 1.93%) in ovary-intact *Oprk1*-*Cre*/tdTomato reporter rats in the prepubertal period (3 weeks of age), whereas *tdTomato* expression was found in most *Tac3*-expressing cells (92.14 ± 1.12%) in OVX + low E2 *Oprk1*-*Cre*/tdTomato reporter rats in adulthood (9–10 weeks of age, Fig. S2e). The number of ARC *Tac3*- and *tdTomato*-coexpressing cells in *Oprk1*-*Cre*/tdTomato reporter rats in prepubertal period was significantly lower than that in adulthood (*t*_(4)_ = 11.34, *p* < 0.01, Student’s *t*-test), although the total number of *Tac3*-expressing cells was comparable between the prepubertal and adult groups (*t*_(4)_ = 1.13, *p* = 0.32, Student’s *t*-test). On the other hand, many *tdTomato*-expressing cells without *Tac3* expression were observed in these regions, consistent with our previous study showing that *Oprk1* expression was found in both KNDy and non-KNDy neurons in the ARC of female rats^25^.

### *Kiss1*-floxed*/Oprk1-Cre *female rats showed puberty onset, the estrous cycle, and fertility, whereas *Kiss1-*floxed*/Kiss1-Cre* female rats lacked puberty onset

**Figure 2:**
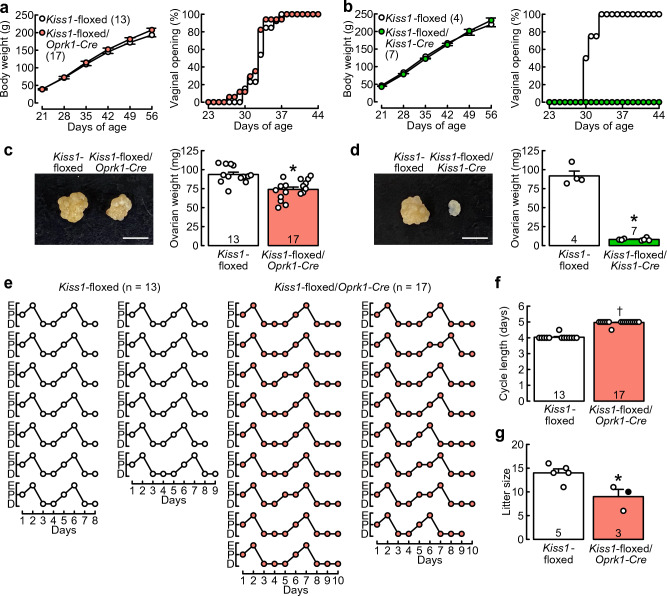
*Kiss1*-floxed/*Oprk1-Cre* female rats showed puberty onset, estrous cycles, and fertility, whereas *Kiss1*-floxed/*Kiss1-Cre* female rats lacked puberty onset. Growth curves and puberty onset in *Kiss1*-floxed/*Oprk1-Cre* female rats and their counterpart Cre(−)/*Kiss1*-floxed female rats (**a**) or *Kiss1*-floxed/*Kiss1-Cre* female rats and their counterpart Cre(−)/*Kiss1*-floxed female rats (**b**). Body weights are the means ± SEM. Numbers in parentheses indicate the number of animals used. The timing of vaginal opening as an external sign of puberty onset is expressed as a percentage of the total number of animals in each group. Representative photographs of ovaries and ovarian weights of *Kiss1*-floxed/*Oprk1-Cre* rats and their counterpart Cre(−)/*Kiss1*-floxed rats (**c**) or *Kiss1*-floxed/*Kiss1-Cre* rats and their counterpart Cre(−)/*Kiss1*-floxed rats (**d**). Scale bars, 5 mm. Open circles indicate the individual data. (**e**) Individual data of estrous cycles from the *Kiss1*-floxed/*Oprk1-Cre* group and Cre(−)/*Kiss1*-floxed control group. E, estrus; P, proestrus; and D, diestrus. (**f**) Cycle length (on average of two consecutive cycles) of *Kiss1*-floxed/*Oprk1-Cre* and Cre(−)/*Kiss1*-floxed groups. A dagger indicates a statistically significant difference (*p* < 0.05) between the groups based on the Wilcoxon rank sum test. Open circles indicate the individual data. (**g**) Litter sizes of *Kiss1*-floxed/*Oprk1-Cre* and Cre(−)/*Kiss1*-floxed female rats, which were mated with Iar:Wistar-Imamichi stud male rats. Values are the means ± SEM. Open (pups were delivered on gestational Day 22) and closed (pups were delivered on gestational Day 23) circles indicate the individual data. Numbers in (or on) each column indicate the number of animals used. Asterisks indicate statistically significant differences (*p* < 0.05) between the groups based on Student’s *t*-test.

*Kiss1*-floxed/*Oprk1-Cre* female rats and *Kiss1*-floxed*/Kiss1-Cre* female rats showed normal growth, similar to that of Cre(−)/*Kiss1*-floxed control rats (Fig. 2a,b). Two-way ANOVA revealed a significant main effect of days of age on the body weight (*Kiss1*-floxed/*Oprk1-Cre*, *F*_(5,140)_ = 3536.12, *p* < 0.01; *Kiss1*-floxed/*Kiss1-Cre*, *F*_(5,45)_ = 1590.38, *p* < 0.01), a significant interaction effect between the genotype and days of age on the body weight (*Kiss1*-floxed/*Oprk1-Cre*, *F*_(5,140)_ = 6.78, *p* < 0.01; *Kiss1*-floxed/*Kiss1-Cre*, *F*_(5,45)_ = 4.41, *p* < 0.01), and no significant differences in body weights between the genotypes at each time point. *Kiss1*-floxed/*Oprk1-Cre* rats and Cre(−)/*Kiss1*-floxed control rats showed vaginal opening (VO) between 27 and 38 days of age (Fig. 2a), and no significant difference was found in the occurrence probability of VO between *Kiss1*-floxed/*Oprk1-Cre* rats and Cre(−)/*Kiss1*-floxed control rats (*χ*^2^ = 0.8, *p* = 0.4, log-rank test). In contrast, *Kiss1*-floxed/*Kiss1-Cre* rats showed no VO until 56 days of age, whereas Cre(−)/*Kiss1*-floxed control rats showed VO between 30 and 33 days of age (Fig. 2b), and there was a significant difference in the occurrence probability of VO between *Kiss1*-floxed/*Kiss1-Cre* rats and Cre(−)/*Kiss1*-floxed control rats (*χ*^2^ = 12.2, *p* < 0.01, log-rank test).

The ovaries of *Kiss1*-floxed/*Oprk1-Cre* and *Kiss1*-floxed/*Kiss1-Cre* rats were smaller than those of Cre(−)/*Kiss1*-floxed control rats, as shown in photographs of the ovaries obtained from representative animals of each genotype (Fig. 2c,d). The ovarian weights in *Kiss1*-floxed/*Oprk1-Cre* rats (77.88 ± 3.19% of the control) and *Kiss1*-floxed/*Kiss1-Cre* rats (8.83 ± 0.61% of the control) were significantly lower than those in Cre(−)/*Kiss1*-floxed control rats (*Kiss1*-floxed/*Oprk1-Cre*, *t*_(28)_ = 4.46, *p* < 0.01; *Kiss1*-floxed/*Kiss1-Cre*, *t*_(9)_ = 17.94, *p* < 0.01, Student’s *t*-test; Fig. 2c,d). Most (12 out of 13) Cre(−)/*Kiss1*-floxed control rats exhibited 4-day (on average of two consecutive estrous cycles) estrous cycles, and remaining one showed a 4-day and 5-day estrous cycle, so that the average value was 4.5-day (Fig. 2e). Notably, most (16 out of 17) *Kiss1*-floxed/*Oprk1-Cre* rats exhibited 5-day (on average) estrous cycles with a 1-day longer diestrus than controls, and remaining one showed a 4-day and 5-day estrous cycle, so that the average value was 4.5-day (Fig. 2e). As a result, the length of the estrous cycle was significantly longer in *Kiss1*-floxed/*Oprk1-Cre* rats than in Cre(−)/*Kiss1*-floxed control rats (*p* < 0.01, Wilcoxon rank sum test, Fig. 2f).

Two out of three *Kiss1*-floxed/*Oprk1-Cre* female rats and all five Cre(−)/*Kiss1*-floxed female rats that mated with wild-type stud male rats at the proestrous stage exhibited spontaneous delivery at gestational Day 22. One remaining *Kiss1*-floxed/*Oprk1-Cre* female rat exhibited spontaneous delivery one day later. The litter size in *Kiss1*-floxed/*Oprk1-Cre* rats was significantly smaller than that in Cre(−)/*Kiss1*-floxed control rats (*t*_(6)_ = 3.17, *p* = 0.02, Student’s *t*-test, Fig. 2g).

### *Kiss1* deletion in cells expressing or having expressed *Oprk1* reduced the number of ARC and AVPV *Kiss1*-expressing cells

**Figure 3:**
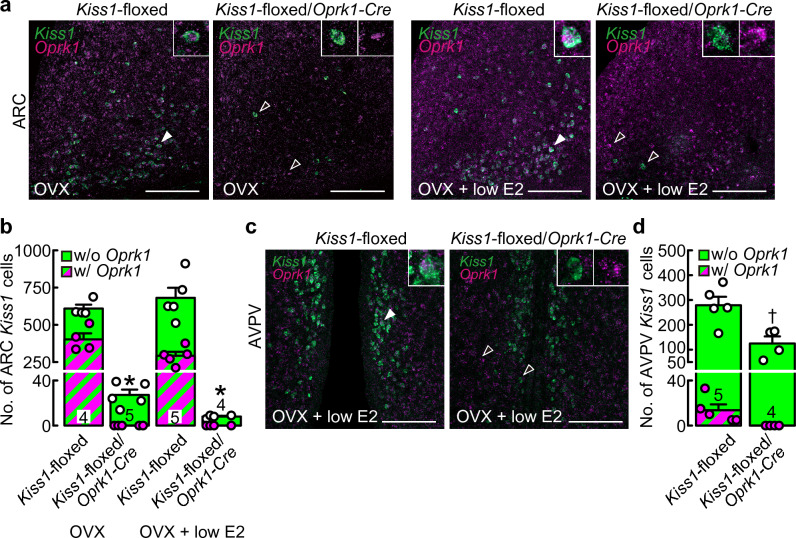
*Kiss1* deletion in cells expressing or having expressed *Oprk1* reduced the number of ARC and AVPV *Kiss1*-expressing cells. (**a**) *Kiss1-*expressing (green) and *Oprk1*-expressing (magenta) cells in the ARC of representative ovariectomized (OVX) or OVX and treated with a negative feedback level of estradiol-17β (OVX + low E2) *Kiss1*-floxed/*Oprk1-Cre* rats and their counterpart Cre(−)/*Kiss1*-floxed rats. (**b**) The numbers of *Kiss1*-expressing (green) or *Kiss1*- and *Oprk1*-coexpressing (striped) cells in the ARC of OVX or OVX + low E2 *Kiss1*-floxed/*Oprk1-Cre* rats and Cre(−)/*Kiss1*-floxed control rats. Asterisks indicate a statistically significant main effect of the genotype (*p* < 0.05) based on two-way ANOVA. (**c**) *Kiss1-*expressing (green) and *Oprk1*-expressing (magenta) cells in the AVPV of representative OVX + low E2 *Kiss1*-floxed/*Oprk1-Cre* rats and their counterpart Cre(−)/*Kiss1*-floxed rats. The insets indicate representative *Kiss1*- and *Oprk1*-coexpressing cells, indicated by the solid white arrowheads, or *Kiss1*- or *Oprk1*-expressing cells, indicated by the open white arrowheads. Scale bars, 100 μm. (**d**) The numbers of *Kiss1*-expressing (green) or *Kiss1*- and *Oprk1*-coexpressing (striped) cells in the AVPV of OVX + low E2 *Kiss1*-floxed/*Oprk1-Cre* rats and Cre(−)/*Kiss1*-floxed control rats. A dagger indicates a statistically significant difference (*p* < 0.05) between the groups based on Student’s *t*-test. Values are the means ± SEM. Circles indicate the individual data of the number of *Kiss1*-expressing (white) or *Kiss1*- and *Oprk1*-coexpressing (magenta) cells. Numbers in (or on) each column indicate the number of animals used.

Double ISH for *Kiss1* and *Oprk1* revealed that a few *Kiss1*-expressing cells without *Oprk1* signals were located in the ARC of OVX *Kiss1*-floxed/*Oprk1-Cre* rats, regardless of E2 treatment, whereas many *Kiss1*-expressing cells with/without *Oprk1* signals were found in the ARC of OVX Cre(−)/*Kiss1*-floxed control rats, regardless of E2 treatment (Fig. 3a). Two-way ANOVA revealed a significant main effect of the genotype, but not E2 treatment, without the interaction effect between the genotype and E2 treatment on the number of ARC *Kiss1*-expressing cells. Specifically, the number of ARC *Kiss1*-expressing cells was significantly lower in *Kiss1*-floxed/*Oprk1-Cre* rats (2.87 ± 0.65% of the control on average with/without low E2 treatment; 8.00 ± 0.71 and 27.20 ± 4.73 cells in *Kiss1*-floxed/*Oprk1-Cre* rats with or without low E2 treatment, respectively) than in Cre(−)/*Kiss1*-floxed control rats (F_(1,14)_ = 246.03, *p* < 0.01, Fig. 3b). Quantitative analysis revealed the absence of *Oprk1* expression in the remaining ARC *Kiss1*-expressing cells in OVX *Kiss1*-floxed/*Oprk1-Cre* rats with/without low E2 treatment, whereas *Oprk1* coexpression was found in Cre(−)/*Kiss1*-floxed control rats (52.58 ± 4.40% of the control on average with/without low E2 treatment).

Double ISH revealed a number of *Kiss1*-expressing cells with/without *Oprk1* expression in the AVPV of OVX + low E2 Cre(−)/*Kiss1*-floxed control rats, while in *Kiss1*-floxed/*Oprk1-Cre* rats, fewer *Kiss1*-expressing cells without *Oprk1* signals were found in the AVPV (Fig. 3c). The number of AVPV *Kiss1*-expressing cells was significantly lower in *Kiss1*-floxed/*Oprk1-Cre* rats (44.75 ± 10.24% of the control) than in Cre(−)/*Kiss1*-floxed control rats (*t*_(7)_ = 3.33, *p* = 0.013, Student’s *t*-test, Fig. 3d). *Oprk1* coexpression was found in 5.26 ± 1.99% of AVPV *Kiss1*-expressing cells in OVX + low E2 Cre(−)/*Kiss1*-floxed control rats.

### OVX *Kiss1*-floxed/*Oprk1-Cre *rats, whose *Kiss1* was deleted in kisspeptin neurons expressing or having expressed *Oprk1*, without E2 treatment showed frequent LH pulses, whereas OVX + low E2 *Kiss1*-floxed/*Oprk1-Cre* rats exhibited profound disruption of LH pulses

**Figure 4:**
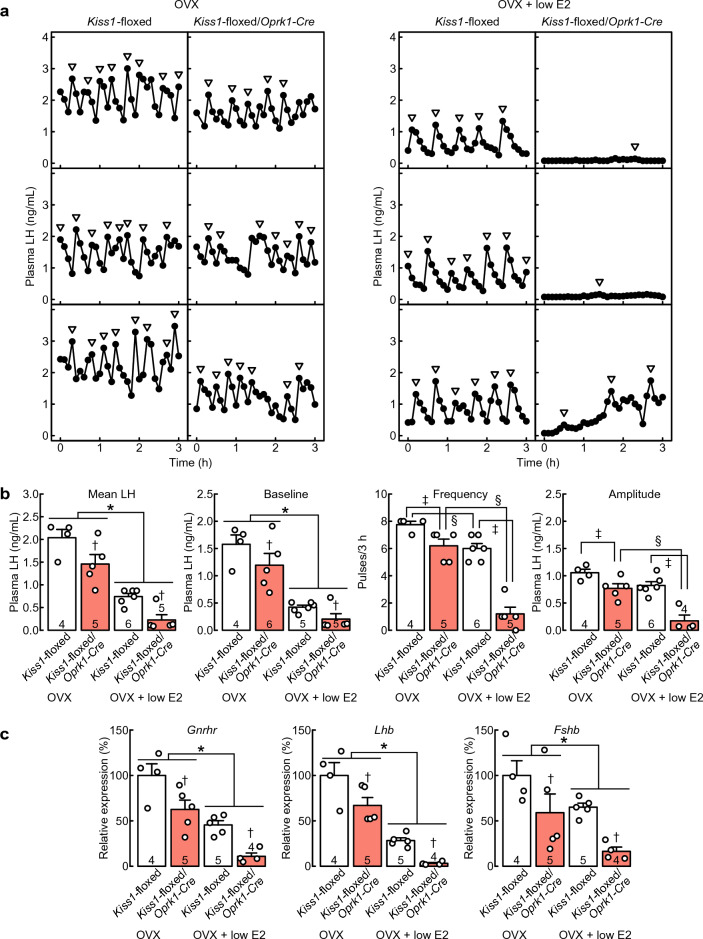
OVX *Kiss1*-floxed/*Oprk1-Cre* rats, whose *Kiss1* was deleted in kisspeptin neurons expressing or having expressed *Oprk1*, without E2 treatment showed frequent luteinizing hormone (LH) pulses, whereas OVX + low E2 *Kiss1*-floxed/*Oprk1-Cre* rats exhibited profound disruption of LH pulses. (**a**) Plasma LH profiles in three representative animals from the OVX or OVX + low E2 *Kiss1*-floxed/*Oprk1-Cre* group and Cre(−)/*Kiss1*-floxed control group. Arrowheads indicate LH pulses identified with the PULSAR computer program. (**b**) Mean LH concentrations and the baseline, frequency, and amplitude of LH pulses in OVX or OVX + low E2 *Kiss1*-floxed/*Oprk1-Cre* rats and Cre(−)/*Kiss1*-floxed control rats. (**c**) Pituitary *Gnrhr*, *Lhb*, and *Fshb* mRNA expression in OVX or OVX + low E2 *Kiss1*-floxed/*Oprk1-Cre* rats and Cre(−)/*Kiss1*-floxed control rats were determined by reverse transcription-quantitative PCR (RT-qPCR). Open circles indicate the individual data. Values are the means ± SEM. Numbers in (or on) each column indicate the number of animals used. Asterisks indicate statistically significant main effects of E2 treatment (*p* < 0.05) based on two-way ANOVA. Daggers indicate statistically significant main effects of the genotype (*p* < 0.05) based on two-way ANOVA. Double daggers indicate statistically significant differences between *Kiss1*-floxed/*Oprk1-Cre* and Cre(−)/*Kiss1*-floxed rats within the OVX or OVX + low E2 groups (*p* < 0.05, the simple main effect of two-way ANOVA). Section signs indicate statistically significant differences between OVX and OVX + low E2 rats within the *Kiss1*-floxed/*Oprk1-Cre* or Cre(−)/*Kiss1*-floxed groups (*p* < 0.05, the simple main effect of two-way ANOVA).

Frequent LH pulses were found in all OVX *Kiss1*-floxed/*Oprk1-Cre* rats and Cre(−)/*Kiss1*-floxed control rats (Fig. 4a). Importantly, pulsatile profiles of LH release were profoundly disrupted in all individual OVX + low E2 *Kiss1*-floxed/*Oprk1-Cre* rats (Fig. 4a), whereas frequent LH pulses were found in all OVX + low E2 Cre(−)/*Kiss1*-floxed control rats (Fig. 4a). Two-way ANOVA revealed significant main effects of E2 treatment and the genotype, without the interaction effects between E2 treatment and the genotype on the mean LH concentration and the baseline of LH pulses (Fig. 4b). Specifically, the mean LH concentration and the baseline of LH pulses were significantly lower in OVX + low E2 rats than in OVX rats (E2 treatment as the main effect; mean LH, *F*_(1,16)_ = 74.36, *, *p* < 0.01; baseline, *F*_(1,16)_ = 59.58, *, *p* < 0.01) and were significantly lower in *Kiss1*-floxed/*Oprk1-Cre* rats than in Cre(−)/*Kiss1*-floxed control rats (genotype as the main effect; mean LH, *F*_(1,16)_ = 13.96, †, *p* < 0.01; baseline, *F*_(1,16)_ = 4.71, †, *p* = 0.045). Two-way ANOVA revealed significant main effects of E2 treatment and the genotype and significant interaction effects between E2 treatment and the genotype on the frequency (E2 treatment,* F*_(1,16)_ = 62.21, *p* < 0.01; genotype, *F*_(1,16)_ = 55.05, *p* < 0.01; E2 treatment × genotype, *F*_(1,16)_ = 14.42, *p* < 0.01) and amplitude of LH pulses (E2 treatment,* F*_(1,15)_ = 24.93, *p* < 0.01; genotype, *F*_(1,15)_ = 31.80, *p* < 0.01; E2 treatment × genotype, *F*_(1,15)_ = 4.76, *p* = 0.046). Specifically, as shown in Fig. 4b, E2 significantly suppressed the frequency of LH pulses in both *Kiss1*-floxed/*Oprk1-Cre* rats (§, *p* < 0.01) and Cre(−)/*Kiss1*-floxed control rats (§, *p* = 0.01) and the amplitude of LH pulses in *Kiss1*-floxed/*Oprk1-Cre* rats (§, *p* < 0.01). In addition, the frequency and amplitude of LH pulses were significantly lower in *Kiss1*-floxed/*Oprk1-Cre* rats than in Cre(−)/*Kiss1*-floxed control rats under OVX (frequency, ‡, *p* = 0.03; amplitude, ‡, *p* = 0.03) and OVX + low E2 (frequency, ‡, *p* < 0.01; amplitude, ‡, *p* < 0.01) conditions.

Figure 4c shows the relative *Gnrhr, Lhb*, and *Fshb* mRNA expression levels in the anterior pituitary gland in *Kiss1*-floxed/*Oprk1-Cre* rats and Cre(−)/*Kiss1*-floxed control rats. Two-way ANOVA revealed significant main effects of E2 treatment and the genotype, without the interaction effects between E2 treatment and the genotype on pituitary *Gnrhr, Lhb*, and *Fshb* mRNA expression (Fig. 4c). Specifically, *Gnrhr, Lhb*, and *Fshb* mRNA expression levels were significantly lower in OVX + low E2 rats than in OVX rats (E2 treatment as the main effect; *Gnrhr, F*_(1,14)_ = 37.65, *, *p* < 0.01; *Lhb*,* F*_(1,14)_ = 69.54, *, *p* < 0.01; *Fshb, F*_(1,14)_ = 7.62, *, *p* = 0.015) and in *Kiss1*-floxed/*Oprk1-Cre* rats than in Cre(−)/*Kiss1*-floxed control rats (genotype as the main effect; *Gnrhr, F*_(1,14)_ = 17.31, †, *p* < 0.01; *Lhb*, *F*_(1,14)_ = 12.78, †, *p* < 0.01; *Fshb, F*_(1,14)_ = 10.18, †, *p* < 0.01).

### High E2 treatment induced a surge-like increase in LH in OVX *Kiss1*-floxed/*Oprk1-Cre *rats, but the LH surge was attenuated by the conditional *Kiss1* deletion in cells expressing or having expressed* Oprk1*

**Figure 5:**
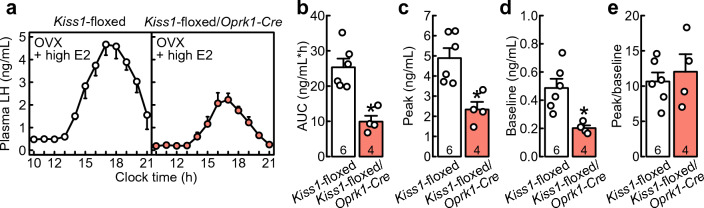
Treatment with a proestrous level of E2** (**high E2) induced a surge-like increase in LH in OVX *Kiss1*-floxed/*Oprk1-Cre* rats, but the LH surge was attenuated by the conditional *Kiss1* deletion in cells expressing or having expressed *Oprk1*. (**a**) Mean plasma LH profiles of OVX + high E2 *Kiss1*-floxed/*Oprk1-Cre* rats and Cre(−)/*Kiss1*-floxed control rats. The area under the curve of the afternoon LH surge (**b**), the peak levels of the LH surge (**c**), the baseline LH levels (**d**), and the ratios of the peak levels of the LH surge to the baseline LH levels (**e**) in OVX + high E2 *Kiss1*-floxed/*Oprk1-Cre* rats and Cre(−)/*Kiss1*-floxed control rats. Values are the means ± SEM. Numbers in each column indicate the number of animals used. An asterisk indicates a statistically significant difference (*p* < 0.05) between the groups based on Student’s *t*-test.

Both OVX + high E2 *Kiss1*-floxed/*Oprk1-Cre* rats and Cre(−)/*Kiss1*-floxed control rats showed an LH surge in the afternoon (Fig. 5a), while the area under the curve (AUC) of plasma LH levels in OVX + high E2 *Kiss1*-floxed/*Oprk1-Cre* rats was significantly lower than that in Cre(−)/*Kiss1*-floxed control rats (*t*_(8)_ = 4.60, *p* < 0.01, Fig. 5b). The peak levels of LH surge (*t*_(8)_ = 3.70, *p* < 0.01, Fig. 5c) and the baseline LH levels (*t*_(8)_ = 3.44, *p* < 0.01, Fig. 5d) were significantly lower in OVX + high E2 *Kiss1*-floxed/*Oprk1-Cre* rats than in Cre(−)/*Kiss1*-floxed control rats. On the other hand, the ratios of the peak levels of LH surge to the baseline LH levels were comparable between the groups (*t*_(8)_ = 0.54, *p* = 0.60, Student’s *t*-test, Fig. 5e).

### *Kiss1*-floxed/*Kiss1-Cre *rats exhibited few ARC *Kiss1*-expressing cells and complete suppression of LH pulses

**Figure 6:**
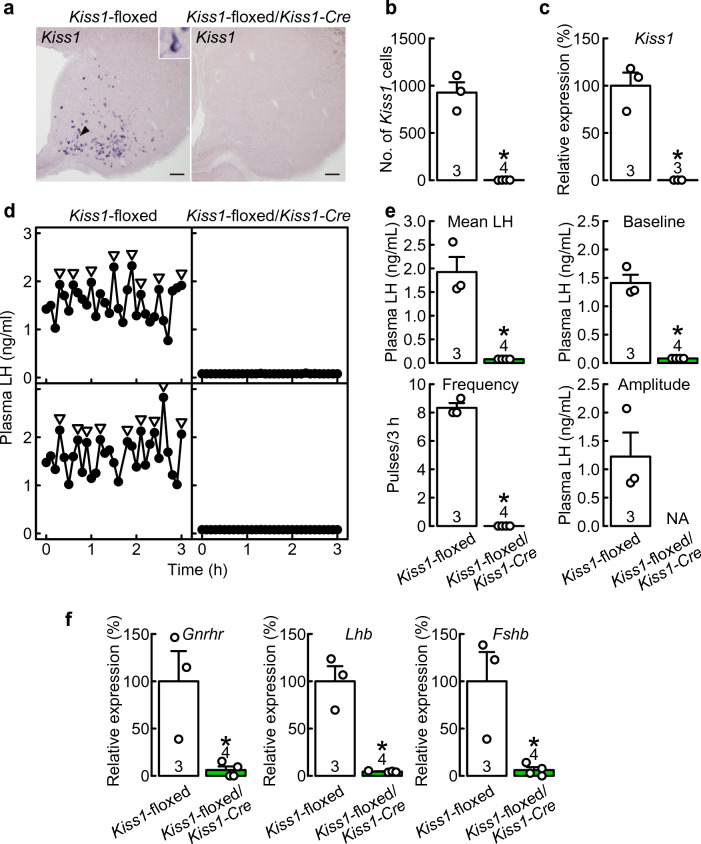
*Kiss1*-floxed/*Kiss1-Cre* rats exhibited few ARC *Kiss1*-expressing cells and complete suppression of LH pulses. (**a**) *Kiss1*-expressing cells in the ARC of representative OVX *Kiss1*-floxed/*Kiss1-Cre* rats and Cre(−)/*Kiss1*-floxed control rats. (**b**) The numbers of *Kiss1*-expressing cells in the ARC of OVX *Kiss1*-floxed/*Kiss1-Cre* rats and Cre(−)/*Kiss1*-floxed control rats. (**c**) ARC *Kiss1* mRNA expression in OVX *Kiss1*-floxed/*Kiss1-Cre* rats and Cre(−)/*Kiss1*-floxed control rats. The ARC *Kiss1* mRNA expression was determined by RT-qPCR. (**d**) Plasma LH profiles of two representative OVX *Kiss1*-floxed/*Kiss1-Cre* rats and Cre(−)/*Kiss1*-floxed control rats. Arrowheads indicate LH pulses identified with the PULSAR computer program. (**e**) Mean LH concentrations and the baseline, frequency, and amplitude of LH pulses in OVX *Kiss1*-floxed/*Kiss1-Cre* rats and Cre(−)/*Kiss1*-floxed rats. (**f**) Pituitary *Gnrhr*, *Lhb*, and *Fshb* mRNA expression in OVX *Kiss1*-floxed/*Kiss1-Cre* rats and Cre(−)/*Kiss1*-floxed rats were determined by RT-qPCR. Values are the means ± SEM. Open circles indicate the individual data. Numbers in (or on) each column indicate the number of animals used. Asterisks indicate statistically significant differences (*p* < 0.05) between the groups based on Student’s *t*-test. NA, not applicable, as no LH pulses were detected in OVX *Kiss1*-floxed/*Kiss1-Cre* rats.

OVX *Kiss1*-floxed/*Kiss1-Cre* rats exhibited few ARC *Kiss1*-expressing cells, whereas many *Kiss1*-expressing cells were detected in the ARC of Cre(−)/*Kiss1*-floxed control rats (Fig. 6a). The number of *Kiss1*-expressing cells was significantly lower in the ARC of *Kiss1*-floxed/*Kiss1-Cre* rats than in Cre(−)/*Kiss1*-floxed control rats (*t*_(5)_ = 10.17, *p* < 0.01, Student’s *t*-test, Fig. 6b). The relative *Kiss1* mRNA expression level in the ARC was significantly lower in OVX *Kiss1*-floxed/*Kiss1-Cre* rats (0.18 ± 0.005% of the control, *p* < 0.01, Student’s *t*-test, Fig. 6c) than in OVX *Cre*(−)/*Kiss1*-floxed control rats.

The plasma LH levels were undetectable, and no LH pulses were found in OVX *Kiss1*-floxed/*Kiss1-Cre* rats, whereas frequent LH pulses were found in OVX Cre(−)/*Kiss1*-floxed control rats (Fig. 6d). The mean LH concentration (*t*_(5)_ = 6.91, *p* < 0.01, Student’s *t*-test) and the baseline (*t*_(5)_ = 10.94, *p* < 0.01, Student’s *t*-test) and frequency of LH pulses (*t*_(5)_ = 29.88, *p* < 0.01, Student’s *t*-test) were significantly lower in *Kiss1*-floxed/*Kiss1-Cre* rats than in Cre(−)/*Kiss1*-floxed control rats (Fig. 6e). Of note, the amplitude of LH pulses in *Kiss1*-floxed/*Kiss1-Cre* rats was not available because no LH pulses were detected in OVX *Kiss1*-floxed/*Kiss1-Cre* rats.

Figure 6f shows the relative *Gnrhr, Lhb*, and *Fshb* mRNA expression levels in the anterior pituitary gland in *Kiss1*-floxed/*Kiss1-Cre* rats and Cre(−)/*Kiss1*-floxed control rats. *Gnrhr* (*t*_(5)_ = 3.46, *p* = 0.017, Student’s *t*-test)*, Lhb* (*t*_(5)_ = 7.14, *p* < 0.01, Student’s *t*-test), and *Fshb* (*t*_(5)_ = 3.60, *p* = 0.016, Student’s *t*-test) mRNA expression levels were significantly lower in *Kiss1*-floxed/*Kiss1-Cre* rats than in Cre(−)/*Kiss1*-floxed control rats (Fig. 6f).

## Discussion

The present study demonstrated that *Kiss1*-floxed/*Oprk1-Cre* female rats, in which the *Kiss1* gene was deleted in KNDy and AVPV kisspeptin neurons expressing or having expressed *Oprk1*, showed profound disruption of LH pulses in the presence of a diestrous level of E2 and a reduction in the AUC of the E2-induced LH surge. These results suggest that the *Kiss1*-deleted kisspeptin neurons in both the ARC and AVPV have a physiological role in maintaining normal reproductive function. Indeed, ovary-intact *Kiss1*-floxed/*Oprk1-Cre* female rats showed a 5-day estrous cycle (vs. a 4-day estrous cycle in Cre(−)/*Kiss1*-floxed control rats), a lower ovarian weight, and a smaller number of pups compared to Cre(−)/*Kiss1*-floxed controls. These findings suggest that *Kiss1* expression in ARC and AVPV kisspeptin neurons, which are expressing or have expressed *Oprk1,* is likely needed to fully generate the GnRH/LH pulse and surge under physiological estrogen conditions. It is speculated that the estrogen-dependent suppression of LH pulses and LH surge attenuation may have caused a lower ovarian weight and a smaller number of pups in ovary-intact *Kiss1*-floxed/*Oprk1-Cre* female rats than in Cre(−)/*Kiss1*-floxed control rats. This is the first report to show that kisspeptin neurons receiving direct Dyn-KOR signaling are needed to fully generate the GnRH/LH pulse and estrogen-induced GnRH/LH surge in OVX female rats under E2 replacement regimens that mimic diestrous and proestrous levels of estrogen, respectively. Notably, the current ovary-intact *Kiss1*-floxed/*Oprk1-Cre* female rats may have maintained effective levels of LH pulses and surges to maintain ovarian function, estrous cycles and fertility even though a longer estrous cycle, a lower ovarian weight, and a smaller number of pups than the wild-type rats.

Importantly, apparent LH pulses were observed in the current OVX *Kiss1*-floxed/*Oprk1-Cre* rats without estrogen replacement, suggesting that the remaining small portion of KOR-negative KNDy neurons are sufficient to maintain GnRH/gonadotropin pulse generation, at least in the estrogen-free condition. This notion is consistent with our previous study showing that the rescue of 3–50% of ARC KNDy neurons resulted in the restoration of GnRH/LH pulses in OVX global *Kiss1* KO rats^6^. Indeed, pituitary *Gnrhr*, *Lhb*, and *Fshb* mRNA expression levels, which largely correlate with GnRH/gonadotropin pulses, in OVX *Kiss1*-floxed/*Oprk1-Cre* rats seemed to be comparable to OVX *Kiss1*-floxed control rats treated with low E2. Therefore, we speculate that the remaining KOR-negative KNDy neurons may synchronize with "*Kiss1*-deficient NDy" neurons in *Kiss1*-floxed/*Oprk1-Cre* female rats via mutual contact among KNDy and "*Kiss1*-deficient NDy" neurons through gap junctions and/or glial connections. Indeed, our previous study showed that gap junction inhibitors blocked synchronized activities among mouse KNDy neurons in vitro^38^, and Moore et al.^9^ and Han et al.^35^ showed using in vivo Ca^2+^ imaging at a single-cell resolution that all recorded KNDy neurons exhibited synchronous activation prior to pulsatile LH release in female and male mice, respectively. Furthermore, Liu et al.^39^ showed that KNDy neurons can generate synchronized episodic activity even in *Kiss1* KO mice. A recent study by Kauffman and colleagues showed that *Kiss* KORKO mice, in which KOR signaling in *Kiss1* neurons was disrupted, exhibited apparent LH pulses in males, normal attainment of puberty in both sexes, and fertility in females^32^. In addition, previous studies showed that both sexes of global *Oprk1* KO mice^40–42^ and global *Pdyn* KO mice^43, 44^ were fertile. In this context, other opioid receptor signaling pathways, such as enkephalin-δ-opioid receptor (DOR) and β-endorphin-μ-opioid receptor (MOR), may be able to compensate for the lack of KOR signaling in KNDy neurons to maintain GnRH pulse generation. In support of this notion, central DOR and MOR antagonism rescued frequent LH pulses in female rats under glucoprivation^45, 46^.

The low E2 treatment disrupted the LH pulse in the current OVX *Kiss1*-floxed/*Oprk1-Cre* rats. This could be simply due to estrogen-dependent suppression of ARC *Kiss1* expression in remaining KOR-negative KNDy neurons, as the E2 treatment tended to reduce the number of ARC *Kiss1*-expressing cells in OVX *Kiss1*-floxed/*Oprk1-Cre* rats. Indeed, the diestrous level of E2 caused a reduction of *Gnrhr*, *Lhb*, and *Fshb* mRNA expression levels in the pituitary of OVX *Kiss1*-floxed/*Oprk1-Cre* rats. In addition, it is tempting to speculate that the estrogen-dependent LH pulse disruption in *Kiss1*-floxed/*Oprk1-Cre* rats may be due to estrogen-dependent activation of some inhibitory signaling pathway(s). In support of this, our previous studies showed that the suppression of LH pulses by fasting or glucoprivation was dependent on the negative feedback level of E2 in OVX rats^47, 48^ and that central DOR or MOR antagonism restored LH pulses in OVX + low E2 rats under glucoprivation^45, 46^. Further studies are required to identify the inhibitory signaling pathway(s) activated by the diestrous level of E2 to suppress GnRH/LH pulse generation in *Kiss1*-floxed/*Oprk1-Cre* rats.

The present study showed that *Oprk1* expression in KNDy neurons increased after the peripubertal period, as *Oprk1*-driven Cre-activated *tdTomato* expression was found in approximately 20% of ARC *Tac3* (a marker of KNDy neurons)-expressing cells at 3 weeks of age (prepubertal period) and 92% of ARC *Tac3*-expressing cells in adulthood. These findings suggest that *Kiss1* may have been knocked out in only 20% of KNDy neurons in *Kiss1*-floxed/*Oprk1-Cre* female rats at the onset of puberty, which may explain why the timing of puberty onset in the current ovary-intact *Kiss1*-floxed/*Oprk1-Cre* female rats was comparable to that in Cre(−)/*Kiss1*-floxed control rats. It is speculated that after puberty onset, the *Kiss1* gene may be gradually knocked out as *Oprk1* expression gradually increases in KNDy neurons. Indeed, the findings in the current *Oprk1-Cre*-activated tdTomato reporter female rats revealed that approximately 90% of KNDy neurons, at least once (even transiently), expressed *Oprk1* mRNA by adulthood. Thus, the *Kiss1* gene was knocked out in a majority of KNDy neurons in adult *Kiss1*-floxed/*Oprk1-Cre* female rats, consequently resulting in a longer estrous cycle, smaller ovarian weight, and smaller litter size than those in Cre(−)/*Kiss1*-floxed control rats.

The current study suggests that AVPV kisspeptin neurons, which are expressing or have expressed *Oprk1,* are needed to generate a full amount of LH surge because the AUC of the high E2-induced LH surge in *Kiss1*-floxed/*Oprk1-Cre* rats was approximately half of that in Cre(−)/*Kiss1*-floxed control rats. The reduction in the LH surge could be simply due to the reduction in the number of AVPV *Kiss1*-expressing cells (approximately half of the control) in the current *Kiss1*-floxed/*Oprk1-Cre* rats, in which the ratio of *Kiss1* KO in AVPV kisspeptin neurons is largely consistent with a previous study showing *Oprk1* mRNA expression in 50% of AVPV kisspeptin neurons in female mice^32^. On the other hand, it is likely that KOR-negative AVPV kisspeptin neurons still function as the GnRH/LH surge generator because the *Kiss1*-floxed/*Oprk1-Cre* rats showed a surge-like increase in LH levels in the afternoon, and the ratios of the peak levels of LH surge to the baseline LH levels were comparable to those in *Kiss1*-floxed control rats. In support of this notion, previous studies showed that ~ 60% of AVPV kisspeptin neurons were activated at the proestrous and E2-induced LH surge in female rodents^10, 12^.

Our findings should be interpreted in the context of the following limitation. *Kiss1* gene was deleted in > 95% of KNDy neurons and > 50% of AVPV kisspeptin neurons in *Kiss1*-floxed/*Oprk1-Cre* female rats, whereas ISH showed *Oprk1* mRNA expression only in approximately 50% of KNDy neurons and 5% of AVPV kisspeptin neurons in *Kiss1*-floxed controls. In addition, *Oprk1*-*Cre*/tdTomato reporter rats showed many Cre-activated *tdTomato*-expressing cells, that did not coexpress *Oprk1* mRNA, in the ARC, PVN, and SON. These inconsistencies may be due to the detection threshold of ISH for *Oprk1*. Namely, *Oprk1* mRNA expression might be below the threshold for the current *Oprk1* ISH in some *Oprk1*-expressing cells. Improvement of the methodology to detect *Oprk1* mRNA may solve the inconsistency between the *Oprk1* expression ratio in ARC and AVPV kisspeptin neurons and the *Kiss1* KO rate in *Kiss1*-floxed/*Oprk1-Cre* female rats and between the number of Cre-activated *tdTomato*-expressing cells and *Oprk1*-expressing cells in *Oprk1-Cre*/tdTomato reporter rats.

In conclusion, the present study demonstrated that kisspeptin neurons, which are expressing and have expressed *Oprk1*, in both the ARC and AVPV had a role in maintaining GnRH pulse and surge generation in female rats because the current *Kiss1*-floxed/*Oprk1-Cre* female rats showed profound disruption of LH pulses in the presence of a diestrous level of E2 and a reduction in the peak of the E2-induced LH surge. This suggests that KNDy and AVPV kisspeptin neurons, which are expressing and have expressed *Oprk1,* seem to be needed for normal GnRH/LH pulse generation and for normal peak levels of the estrogen-induced GnRH/LH surge, respectively, to support a normal estrous cycle length and offspring number in ovary-intact female rats. Another important finding of the study is that *Kiss1*-floxed/*Oprk1-Cre* rats, in which *Kiss1* was expressed only in KOR-negative kisspeptin neurons, were still fertile and maintained the GnRH/gonadotropin pulse-generating mechanism, especially under the steroid-free condition.

## Methods

### Animals

*Oprk1-Cre*/tdTomato reporter rats were generated by crossing newly generated *Oprk1-Cre* rats (see below for details) and tdTomato reporter rats [LE-Tg(Gt(ROSA)26Sor-CAG-tdTomato)24Jfhy rats^37^, supplied by the National BioResource Project—Rat, Kyoto University, Kyoto, Japan (NBRP Rat No. 0734) with support in part by the National BioResource Project of the Ministry of Education, Culture, Sports, Science and Technology (MEXT), Tokyo, Japan]. *Kiss1*-floxed/*Oprk1-Cre* rats were generated by crossing *Oprk1-Cre* rats and *Kiss1*-floxed rats (Rat Genome Database identification (RGD ID): 125097497)^6^. The resulting *Kiss1*-floxed/*Oprk1-Cre* (specifically, *Kiss1*^fl/fl^/*Oprk1*^Cre+^) female rats and Cre(−)/*Kiss1*-floxed (specifically, *Kiss1*^fl/fl^/*Oprk1*^Cre-^) female control rats were used. *Kiss1*-floxed/*Kiss1-Cre* rats were generated by crossing *Kiss1-Cre* rats^36^ and *Kiss1*-floxed rats. The resulting *Kiss1*-floxed/*Kiss1-Cre* (specifically, *Kiss1*^fl/Cre+^) female rats and Cre(−)/*Kiss1*-floxed (specifically, *Kiss1*^fl/Cre-^) female control rats were used. Wild-type Iar:Wistar-Imamichi male rats (RGD ID: 125097496, Institute for Animal Reproduction, Kasumigaura, Japan) were used for the mating test. All rats were maintained in a room with a 14:10-h light/dark cycle (lights on at 05:00 h) at 22 ± 3 °C and had free access to food (CE-2; CLEA Japan, Tokyo, Japan) and water. Female rats were housed by groups of 2–4 animals per cage (if not otherwise specified). Female rats with two consecutive 4- or 5-day estrous cycles, as determined by vaginal smears, were mated overnight with male rats on the day of the proestrus. The resultant pregnant females, as determined by the presence of vaginal plugs, were housed individually. The day when the vaginal plug was found in the morning was designated gestational Day 0, and the day when the newborn litter was found at noon was designated postnatal Day 0. Genotypes were analyzed by PCR with DNA obtained from newborn pups. The primers are listed in Supplementary Table S1 online. The litter size was recorded and adjusted to eight by postnatal Day 5 to minimize the growth variation within and between litters. The pups were weaned on postnatal Day 20 or 21. Animals were subjected to brain, pituitary, ovary, or blood collection at 8–13 weeks of age (adulthood) or 21 days of age (prepubertal period). If not otherwise specified, surgeries were conducted under aseptic conditions and anesthesia with an intraperitoneal injection of a ketamine (27 mg/kg)–xylazine (5.3 mg/kg) mixture, followed by inhalation of isoflurane (1–2% in air). After the surgery, female rats were housed individually. Care of the animals and all experimental procedures performed in the present study were reviewed and approved by the Animal Experiment Committees of Nagoya University and the National Institutes of Natural Sciences. This study was conducted in accordance with the Nagoya University Regulations on Animal Care and Use in Research and ARRIVE guidelines.

### Gene targeting and generation of *Oprk1-Cre* rats

To generate *Oprk1-Cre* rats, a targeting vector was designed to replace the stop codon of the *Oprk1* gene with the *T2A*-*Cre* sequence (see Supplementary Fig. S1 online). The 5’- and 3’-homology arms and *T2A*-*Cre* sequences were inserted into pAAV-MCS3. Production of adeno-associated virus (AAV) type 6 carrying the targeting vector was performed as described previously^49^. A guide RNA targeted around the stop codon of the rat *Oprk1* gene (5′-TAAGCCAGTATGACTAGTCA-3′) was synthesized by and purchased from Integrated DNA Technologies (Coralville, IA). The CRISPR/Cas9 components were introduced by electroporation, and the AAV targeting vector was transfected at the pronuclear stage of rat zygotes collected from Crlj:WI rats (RGD ID: 2312504, Charles River Laboratories Japan, Yokohama, Japan), as described previously^50^. Two-cell stage embryos were transferred to pseudopregnant female rats under isoflurane anesthesia (2–3% in air). Genotypes of the obtained animals were confirmed by PCR and sequencing. The primers for PCR are listed in Supplementary Table S1 online.

### Brain sampling from prepubertal and adult *Oprk1*-*Cre*/tdTomato reporter female rats

Adult female *Oprk1-Cre*/tdTomato reporter rats (n = 3, collected from 3 litters) were OVX and immediately received subcutaneous Silastic tubing (1.57 mm inner diameter; 3.18 mm outer diameter; 25 mm in length; Dow Corning, Midland, MI) filled with E2 (Sigma‒Aldrich, St. Louis, MO) dissolved in peanut oil (Sigma‒Aldrich) at 20 μg/ml for 1 week to produce a negative feedback level of plasma E2 (low E2)^47^. Ovary-intact prepubertal *Oprk1-Cre*/tdTomato reporter rats (n = 3, collected from 2 litters) and OVX + low E2 adult *Oprk1-Cre*/tdTomato reporter rats were deeply anesthetized with sodium pentobarbital (40 mg/kg, Tokyo Chemical Industry, Tokyo, Japan) and then intracardially perfused with phosphate-buffered saline, followed by 4% paraformaldehyde (Sigma‒Aldrich). The brains were immediately removed and postfixed in the same fixative overnight at 4 °C, followed by immersion in 30% sucrose in 0.05 M phosphate buffer until the brains sank at 4 °C. Frozen frontal sections containing the ARC (50-μm thickness) were prepared using a cryostat (CM1800, Leica Biosystems, Wetzlar, Germany). Every fourth ARC section was used for double ISH to visualize *tdTomato*/*Oprk1* or *Tac3*/*tdTomato*. *Tac3* was used as a marker of KNDy neurons because our previous study showed that *Tac3* expression was evident in KNDy neurons before and after puberty onset in female rats^51^.

### Analyses of body weight, puberty onset, and vaginal cyclicity and ovarian sampling from *Kiss1*-floxed/*Oprk1-Cre *and *Kiss1*-floxed*/Kiss1-Cre* female rats

The body weights of *Kiss1*-floxed/*Oprk1-Cre* rats (n = 17) and their counterpart Cre(−)/*Kiss1*-floxed rats (n = 13), as well as *Kiss1*-floxed/*Kiss1-Cre* rats (n = 7) and their counterpart Cre(−)/*Kiss1*-floxed rats (n = 4), were measured every morning after weaning. Female rats were collected from 2–8 litters. VO as a sign of pubertal onset and then the vaginal smear pattern were examined to monitor stages of the estrous cycle at least until 56 days of age. The estrous cycle length was determined for two consecutive estrous cycles of each individual and the mean length of each individual was calculated. Adult female rats were then OVX, and both ovaries were weighed.

### Blood, pituitary, and brain sampling from OVX *Kiss1*-floxed/*Oprk1-Cre *and *Kiss1*-floxed/*Kiss1-Cre* rats

Two weeks after ovariectomy (as described above), OVX *Kiss1*-floxed/*Oprk1-Cre* rats (n = 5) and their counterpart Cre(−)/*Kiss1*-floxed rats (n = 4), as well as *Kiss1*-floxed/*Kiss1-Cre* rats (n = 4) and their counterpart Cre(−)/*Kiss1*-floxed rats (n = 3), were subjected to frequent blood sampling to determine LH pulses. A silicon cannula for blood sampling (inner diameter 0.5 mm and outer diameter 1.0 mm; Shin-Etsu Polymer, Tokyo, Japan) was inserted into the right atrium through the jugular vein 1 day before the onset of blood sampling. Blood samples (100 μL) were collected from freely moving conscious OVX rats every 6 min for 3 h (13:00 to 16:00 h), and plasma samples were collected after the centrifugation at 4 °C. An equivalent volume of rat red blood cells, which were taken from donor rats and diluted with heparinized saline, was replaced through the atrial catheter after each blood collection to keep the hematocrit constant. One day after blood sampling, the brains and anterior pituitary were collected. The brains were processed as described above. Frozen frontal sections containing the ARC (50-μm thickness) were prepared using a cryostat. Every fourth ARC section was used for ISH to visualize *Kiss1* and *Oprk1* in *Kiss1*-floxed/*Oprk1-Cre* rats or *Kiss1* in *Kiss1*-floxed/*Kiss1-Cre* rats. The anterior pituitary was stored at − 80 °C for reverse transcription-quantitative PCR (RT-qPCR) analyses for *Gnrhr, Lhb*, and *Fshb* mRNA expression.

### Blood, pituitary, and brain sampling from E2-treated OVX *Kiss1*-floxed/*Oprk1-Cre* rats

*Kiss1*-floxed/*Oprk1-Cre* rats (n = 5) and their counterpart Cre(−)/*Kiss1*-floxed control rats (n = 6) were OVX (as described above) and immediately received Silastic tubing filled with E2 dissolved in peanut oil at 20 μg/mL for 1 week to serve as OVX + low E2 rats. OVX + low E2 *Kiss1*-floxed/*Oprk1-Cre* rats and Cre(−)/*Kiss1*-floxed control rats were subjected to frequent blood sampling to determine LH pulses as described above. One day after the frequent blood sampling, the estrogen tubing was replaced with another tube containing E2 dissolved in peanut oil at 1000 μg/mL to produce a positive feedback level of plasma E2 (high E2)^6, 52^. Blood samples (100 μL) were collected from *Kiss1*-floxed/*Oprk1-Cre* rats (n = 4) and their counterpart Cre(−)/*Kiss1*-floxed control rats (n = 6) every 1 h from 10:00 to 21:00 h 2 days after the high E2 replacement to determine the afternoon LH surge. One day after the second blood sampling to detect the LH surge, the estrogen tubing was replaced with another tube containing E2 dissolved in peanut oil at 20 μg/mL (low E2). Estrogen tubing replacement was performed under isoflurane anesthesia (2–3% in air). One week later, the brains and anterior pituitary of *Kiss1*-floxed/*Oprk1-Cre* rats (n = 4) and their counterpart Cre(−)/*Kiss1*-floxed control rats (n = 5) were collected and processed as described above. Every fourth ARC section and every second AVPV section were used for double ISH to visualize *Kiss1* and *Oprk1*.

### Brain sampling from OVX *Kiss1*-floxed/*Kiss1-Cre* rats

Two weeks after ovariectomy (as described above), *Kiss1*-floxed/*Kiss1-Cre* rats (n = 3) and Cre(−)/*Kiss1*-floxed rats (n = 3) were decapitated between 13:00 h and 15:00 h. The ARC-median eminence region (approximately 1.8 and 4.0 mm posterior to the bregma) was dissected from the brain with a microblade as previously described^53^.

### RT-qPCR analyses of ARC *Kiss1* mRNA expression and pituitary *Gnrhr, Lhb*, and *Fshb* mRNA expression

Total RNA was extracted from the ARC using ISOGEN (Nippon Gene, Tokyo, Japan). Total RNA was extracted from the fixed hemipituitary by using the RNeasy FFPE kit (QIAGEN) as described previously^6^. cDNA from each sample was synthesized with oligo (deoxythymidine) primers at 37 °C using the high-capacity cDNA reverse transcription kit (Applied Biosystems, Foster City, CA). Gene expression levels were determined using QuantStudio 3 (Applied Biosystems) with the Thunderbird SYBR Green qPCR Mix (TOYOBO, Osaka, Japan) as described previously^38, 52^. The forward and reverse primers for *Kiss1, Gnrhr, Lhb, Fshb,* and *Actb* (encoding β-actin) are listed in Supplementary Table S1 online. The specificity of the amplification products was confirmed by dissociation curve analysis. The relative gene expression levels of *Kiss1, Gnrhr, Lhb,* and *Fshb* were normalized to *Actb*, and the fold changes between the groups were calculated using the 2^-ΔΔCT^ method.

### Double ISH for *tdTomato*/*Oprk1* or *Tac3*/*tdTomato* in the hypothalamus of *Oprk1-Cre*/tdTomato reporter rats and for *Kiss1*/*Oprk1* in the hypothalamus of *Kiss1*-floxed/*Oprk1-Cre* female rats

Double ISH for *tdTomato*/*Oprk1*, *Tac3*/*tdTomato,* or *Kiss1*/*Oprk1* was performed as described previously^54, 55^. Briefly, brain sections were hybridized overnight at 60 °C with a fluorescein isothiocyanate (FITC)-labeled antisense complementary RNA (cRNA) probe and a digoxigenin (DIG)-labeled antisense cRNA probe. The brain sections from *Oprk1-Cre*/tdTomato reporter rats were hybridized with a *tdTomato*-specific FITC-labeled cRNA probe (positions 610–1105, AY678269) and an *Oprk1*-specific DIG-labeled cDNA probe (positions 66–768 and 805–1956; NM_017167) or a *Tac3*-specific FITC-labeled cRNA probe (positions 180–304, NM_019162) and a *tdTomato*-specific DIG-labeled cRNA probe*.* The brain sections from *Kiss1*-floxed/*Oprk1-Cre* and their counterpart Cre(−)/*Kiss1*-floxed control rats were hybridized with a *Kiss1*-specific FITC-labeled antisense cRNA probe (positions 33–349, AY196983) and the *Oprk1*-specific DIG-labeled cDNA probe. The hybridized FITC-labeled probe was detected with a peroxidase (POD)-conjugated anti-FITC antibody (Roche Diagnostics, Indianapolis, IN, RRID: AB_840257) and the TSA Plus Fluorescein System (1:100; Akoya Biosciences, Marlborough, MA), and the hybridized DIG-labeled probe was detected with a POD-conjugated anti-DIG antibody (Roche Diagnostics, RRID: AB_514500), the TSA Plus Biotin Kit (1:100; Akoya Biosciences), and DyLight 594-conjugated streptavidin (Thermo Fisher Scientific, Waltham, MA). The fluorescent images were examined under a fluorescence microscope with ApoTome.2 optical sectioning (Carl Zeiss, Oberkochen, Germany).

The numbers of *Oprk1*- and/or *tdTomato*-expressing cells in the brain sections that underwent double ISH for *tdTomato*/*Oprk1* were counted unilaterally in the ARC (from 1.72 to 4.36 mm posterior to the bregma), PVN (from 0.96 to 1.92 mm posterior to the bregma), and SON (from 1.20 to 1.80 mm posterior to the bregma) according to the rat brain atlas^56^. The numbers of *Tac3*-expressing and *Tac3*- and *tdTomato*-coexpressing cells were counted unilaterally in the ARC. The numbers of *Kiss1*-expressing and *Kiss1*- and *Oprk1*-coexpressing cells in the brain sections from OVX + low E2 *Kiss1*-floxed/*Oprk1-Cre* rats and Cre(−)/*Kiss1*-floxed control rats were counted unilaterally in the ARC and AVPV (from 0.12 mm anterior to 0.60 mm posterior to the bregma). The numbers of *Kiss1*-expressing and *Kiss1*- and *Oprk1*-coexpressing cells in the brain sections from OVX *Kiss1*-floxed/*Oprk1-Cre* rats and Cre(−)/*Kiss1*-floxed control rats were counted unilaterally in the ARC.

The specificity of antisense cRNA probes was verified by control experiments using sense cRNA probes. No signals were found in the sections incubated with the sense cRNA probes.

### ISH for *Kiss1* expression in the ARC of OVX *Kiss1*-floxed/*Kiss1-Cre* rats

Single ISH for *Kiss1* was performed as described previously^10^. Briefly, brain sections were hybridized overnight at 60 °C with a *Kiss1*-specific DIG-labeled antisense cRNA probe (positions 33–349, AY196983). The hybridized probe was detected with an alkaline phosphatase-conjugated anti-DIG antibody (1:1,000; Roche Diagnostics, RRID: AB_2734716) and a chromogen solution (337 μg/mL 4-nitro blue tetrazolium chloride and 175 μg/mL 5-bromo-4-chloro-3-indolyl-phosphate, Roche Diagnostics). The *Kiss1*-expressing cells throughout the ARC were unilaterally counted under a light microscope, BX53 (Olympus, Tokyo, Japan).

### Radioimmunoassay (RIA) and analyses of LH pulse and surge parameters

Plasma LH concentrations were determined by a double-antibody RIA with a rat LH-RIA kit provided by the National Hormone and Peptide Program. The concentrations were expressed in terms of rat LH-RP3. The lowest detectable level in the LH assay was 3.9 pg/tube, and the intra- and interassay coefficients of variation were 4.76% and 7.36% at 34 pg/tube, respectively. LH pulses were identified by the PULSAR computer program^57, 58^. LH pulse parameters, such as the mean LH concentration and the baseline, frequency, and amplitude of LH pulses, were calculated during the 3-h sampling period for each individual and then for the group. For the E2-induced LH surge, LH surge parameters, such as the AUC (from 13:00 to 21:00 h), the peak levels of the LH surge (the highest plasma LH level in the afternoon), the baseline LH levels (mean plasma LH levels at 10:00, 11:00, and 12:00 h), and the ratio of the peak levels of the LH surge to the baseline LH levels were calculated for each individual and then for the group.

### Statistical analysis

Statistical differences in the numbers of ARC *Tac3*-expressing cells and *Tac3*- and *tdTomato*-coexpressing cells between prepubertal and adult *Oprk1-Cre*/tdTomato reporter rats were determined by Student’s *t*-test. Statistical differences in ovarian weights, litter sizes, the number of *Kiss1*-expressing cells in the AVPV, and LH surge parameters between *Kiss1*-floxed/*Oprk1-Cre* rats and Cre(−)/*Kiss1*-floxed rats were determined by Student’s *t*-test. Statistical differences in ovarian weights, the number of *Kiss1*-expressing cells in the ARC, ARC *Kiss1* expression levels, LH pulse parameters, and pituitary *Gnrhr*, *Lhb*, and *Fshb* expression levels between *Kiss1*-floxed/*Kiss1-Cre* rats and Cre(−)/*Kiss1*-floxed rats were determined by Student’s *t*-test. Statistical differences in the timing of VO between *Kiss1*-floxed/*Oprk1-Cre* rats or *Kiss1*-floxed/*Kiss1-Cre* rats and Cre(−)/*Kiss1*-floxed rats were determined by Kaplan‒Meier analysis and the log-rank test. Statistical differences in the lengths of estrous cycles were determined by the Wilcoxon rank sum test. The statistical analyses described above were performed using R version 3.4.2 (https://www.R-project.org/). Statistical differences in body weights between *Kiss1*-floxed/*Oprk1-Cre* or *Kiss1*-floxed/*Kiss1-Cre* rats and Cre(−)/*Kiss1*-floxed rats were determined by two-way ANOVA for repeated measures (main effects, groups and days), followed by analyses of simple main effects. Statistical differences in the number of *Kiss1*-expressing cells in the ARC, LH pulse parameters, and pituitary *Gnrhr*, *Lhb*, and *Fshb* expression levels between OVX *Kiss1*-floxed/*Oprk1-Cre* rats and Cre(−)/*Kiss1*-floxed control rats with/without low E2 treatment were determined by two-way ANOVA (main effects, genotype and E2 treatment), followed by analyses of simple main effects. Two-way ANOVA was performed using SAS OnDemand for Academics (https://welcome.oda.sas.com). Differences were considered statistically significant at *p* < 0.05.

### Supplementary Information

Supplementary Information.

## Data Availability

The datasets generated during and/or analyzed during the current study are available from the corresponding authors on reasonable request.
