# Supplementary material for: Conditional Oprk1-dependent Kiss1 deletion in kisspeptin neurons caused estrogen-dependent LH pulse disruption and LH surge attenuation in female rats
Source: Sci Rep. 2023 Nov 22;13:20495. doi: 10.1038/s41598-023-47222-5 (PMC10665460; doi:10.1038/s41598-023-47222-5)
Supplement: Supplementary file 1 — Supplementary Information. [file 41598_2023_47222_MOESM1_ESM.pdf]

Supplementary Information for

Conditional *Oprk1*-dependent *Kiss1* deletion in kisspeptin neurons caused estrogen-dependent LH pulse disruption and LH surge attenuation in female rats

Mayuko Nagae[1,2], Koki Yamada[1], Yuki Enomoto[1], Mari Kometani[1], Hitomi Tsuchida[1], Arvinda Panthee[1], Miku Nonogaki[1], Nao Matsunaga[1], Marina Takizawa[1], Sena Matsuzaki[1], Masumi Hirabayashi[2], Naoko Inoue[1], Hiroko Tsukamura[1*], and Yoshihisa Uenoyama[1*]

[1]Laboratory of Animal Reproduction, Graduate School of Bioagricultural Sciences, Nagoya University, Nagoya, Aichi 464-8601, Japan

[2]Section of Mammalian Transgenesis, Center for Genetic Analysis of Behavior, National Institute for Physiological Sciences, Okazaki, Aichi 444-8787, Japan

*htsukamura@nagoya-u.jp (H.Tsukamura) and uenoyama@nagoya-u.jp (Y.U.)

Table S1. Primer sequences for the genotyping of *Oprk1-Cre*, *Kiss1-Cre*, and *Kiss1*-floxed rats, screening of *Oprk1-Cre* founder rats, and for the reverse transcription-quantitative PCR (RT-qPCR) analysis for *Kiss1* and *Actb*.

Purpose	Targets	Sequence<br>forward<br>reverse
Genotyping	*Oprk1-Cre* rats	5'-gatgtcattgaatgctccttgc-3'
		5'-ttggctactggcatcatctg-3'
	Kiss1-Cre rats	5'-ccttgtttggggcttatcct-3'
		5'-ttgcccctgtttcactatcc-3'
	Kiss1-floxed rats	5'-tcctgcctgaccttaccaac-3'
		5'-agtaccgattttggcaccag-3'
Screening	*Oprk1-Cre* founder rats	5'-atgtcataccacccacccag-3'
	(5'-PCR)	5'-ccgccgcataaccagtgaaac-3'
	Oprk1-Cre founder rats	5'-cagcaacatttgggccagcta-3'
	(3'-PCR)	5'-ggcctccatctccagaaagt-3'
RT-qPCR	*Kiss1* (NM_181692.1)	5'-agctgctgcttctcctctgt-3'
		5'-aggcttgctctctgcatacc-3'
	Actb (NM_031144.3)	5'-tgtcaccaactgggacgata-3'
		5'-ggggtgttgaaggtctcaaa-3'
	Gnrhr (NM_031038.3)	5'-ccagccttcatgatggtggt-3'
		5'-gggatgatgaacaggcagct-3'
	Lhb (NM_012858.2, NM_001033975.1)	5'-atgagttctgcccagtctgc-3'
		5'-tggggaaggtcacaggtcat-3'
	Fshb (NM_001007597.2)	5'-agctgttgacttacctggcc-3'
		5'-gggtgtttggtctagctggg-3'

Gene symbols and protein names are as follows: *Actb*, β-actin; *Fshb*, FSH β-subunit; *Gnrhr*, GnRH receptor; *Kiss1*, kisspeptin; *Lhb*, LH β-subunit; and *Oprk1*, κ-opioid receptor.

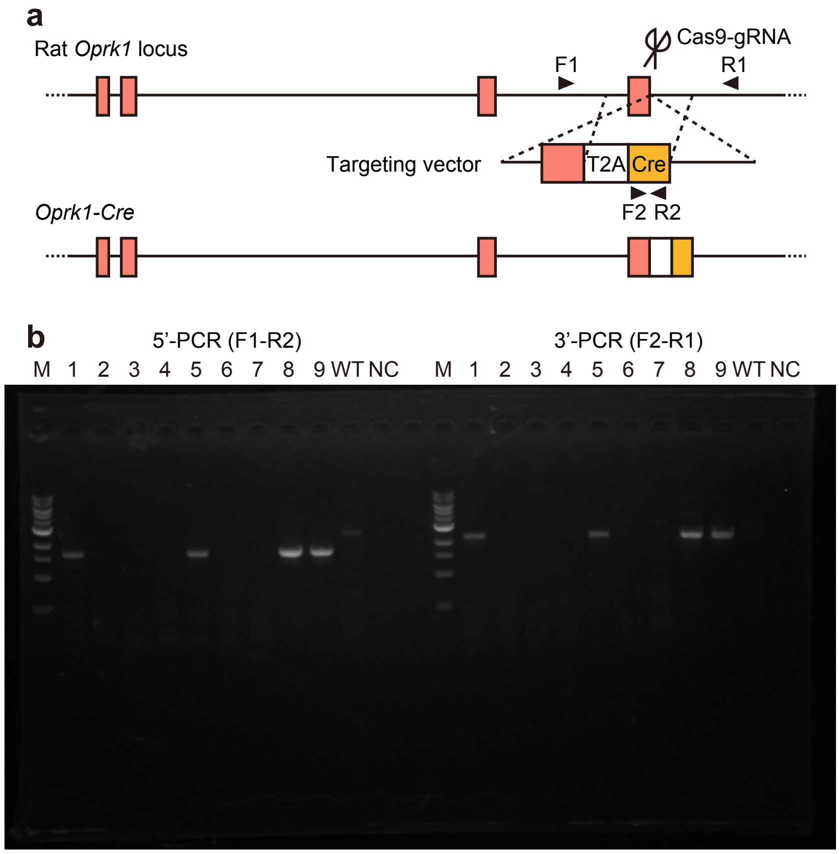

Figure S1. Targeting of the *Oprk1* locus for the generation of *Oprk1-Cre* rats. (**a**) Structure of the wild-type *Oprk1* allele (top), the *Oprk1-Cre* targeting vector (middle), and the targeted *Oprk1* allele (bottom), resulting from replacement recombination at the dotted lines by CRISPR/Cas9 system. (**b**) Screening of founder rats by PCR using primers (solid arrowheads in the panel **a**) located outside the 5' or 3' end of targeting vector and in the *Cre* gene. Primer sets of 5'-PCR (F1 and R2 primers) and 3'-PCR (F2 and R1 primers) generate 1,712 and 2,343 bp products, respectively. The sequences of the primers and guide RNA are shown in Table S1 and main text, respectively. M, DNA marker; WT, wild-type; NC, negative control.

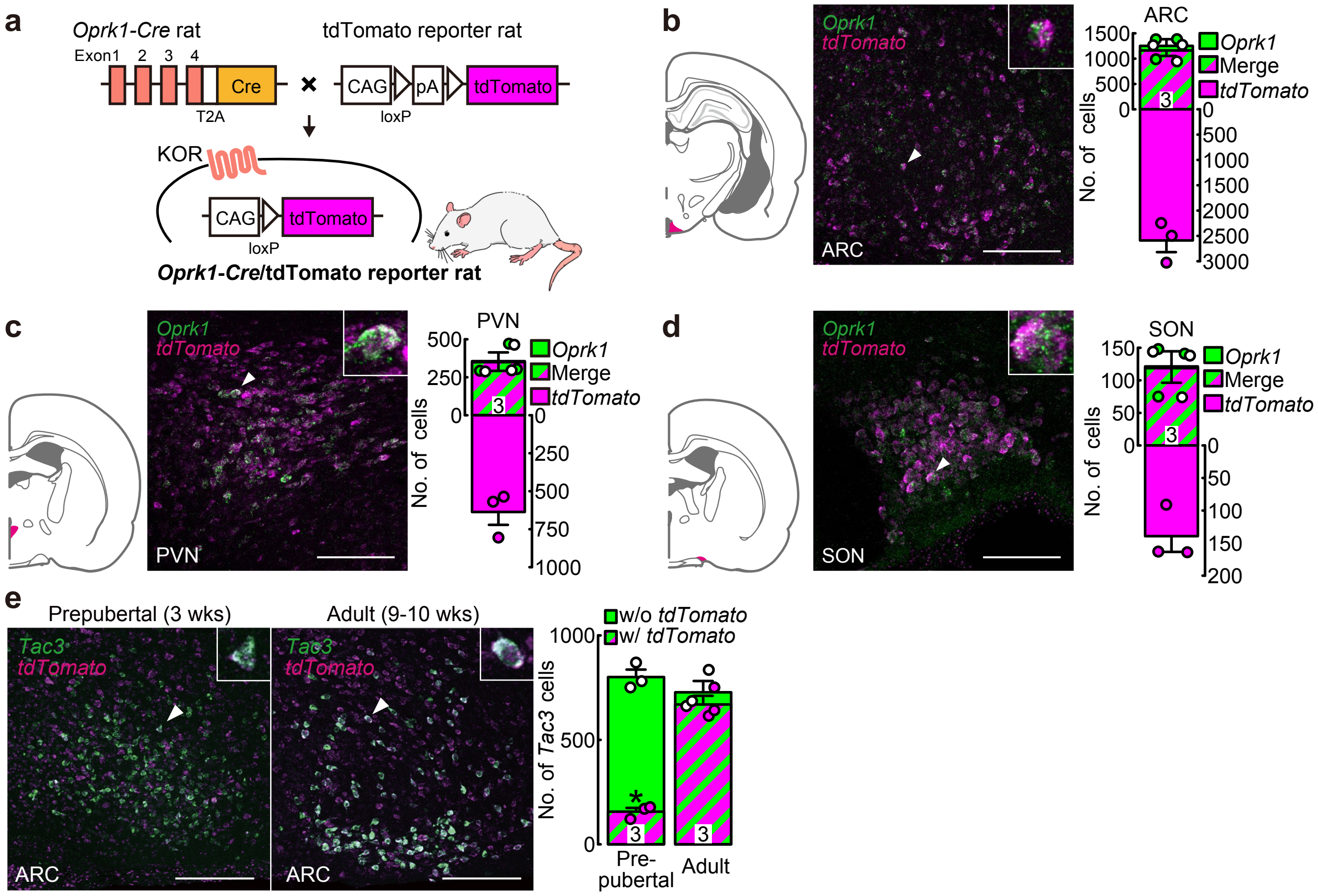

Figure S2. Colocalization of *Oprk1* and *Oprk1-Cre*-activated *tdTomato* mRNA expression in the hypothalamic nuclei, such as the arcuate nucleus (ARC), paraventricular nucleus (PVN), and supraoptic nucleus (SON) and pubertal changes in *Oprk1* expression in KNDy neurons in *Oprk1-Cre*/tdTomato reporter rats. (**a**) Schematic illustration of crossing *Oprk1-Cre* rats with tdTomato reporter rats to obtain *Oprk1-Cre*/tdTomato reporter rats. Schematic illustrations of the brain section showing the ARC (**b**), PVN (**c**), and SON (**d**) highlighted in magenta (left). *Oprk1*-expressing (green) and *Oprk1-Cre*-activated *tdTomato*-expressing (magenta) cells in the aforementioned nuclei of representative a diestrous level of estradiol-17β (E2)-treated ovariectomized (OVX + low E2) *Oprk1-Cre*/tdTomato reporter rats (middle). The insets indicate *Oprk1*- and *tdTomato*-coexpressing cells pointed by the solid white arrowheads at higher magnification. Note that pseudocolors were exchanged between *tdTomato* (detected by TSA Plus Fluorescein System) and *Oprk1* (detected by TSA Plus Biotin Kit and DyLight

594-conjugated streptavidin). The numbers of *Oprk1*-expressing (green), *Oprk1-Cre*-activated *tdTomato*-expressing (magenta), or *Oprk1*- and *tdTomato*-coexpressing (striped) cells in OVX + low E2 *Oprk1-Cre*/tdTomato reporter rats (right). Circles indicate the individual data of the number of *Oprk1*-expressing (green), tdTomato-expressing (magenta), or *Oprk1*- and *tdTomato*-coexpressing (white) cells. (**e**) *Tac3*-expressing (green) and *Oprk1-Cre*-activated *tdTomato*-expressing (magenta) cells in the ARC of representative intact prepubertal (left) and OVX + low E2 adult *Oprk1-Cre*/tdTomato reporter rats (middle). The insets indicate *Tac3*- and *tdTomato*-coexpressing cells pointed by the solid white arrowheads at higher magnification. Scale bars, 100 μm. The numbers of *Tac3*-expressing (green) or *Tac3*- and *tdTomato*-coexpressing (striped) cells in the ARC (right). Values are the means ± SEM. Circles indicate the individual data of the number of *Tac3*-expressing (white) or *Tac3*- and *tdTomato*-coexpressing (magenta) cells. Numbers in each column indicate the number of animals used. An asterisk indicates statistically significant differences ($p < 0.05$) between the groups based on Student's *t*-test.
